# Continuous Exposure to Terrorism during the COVID-19 Pandemic: A Moderated Mediation Model in the Israeli Context

**DOI:** 10.3390/ijerph20042799

**Published:** 2023-02-04

**Authors:** Becky Leshem, Gabriela Kashy-Rosenbaum, Miriam Schiff, Rami Benbenishty, Ruth Pat-Horenczyk

**Affiliations:** 1 Department of Education, Achva Academic College, Beer Tuvia, Arugot 7980400, Israel; 2 Department of Education, Ashkelon Academic College, Ashkelon 7810001, Israel; 3 Paul Baerwald School of Social Work and Social Welfare, Hebrew University of Jerusalem, Jerusalem 9190501, Israel; 4 Department of Education, Universidad Andres Bello, Santiago 7591538, Chile

**Keywords:** continuous traumatic stress, COVID-19, higher education, social support, depression

## Abstract

This study tested the role of perceived social support as a moderating factor in the mediation of COVID-19-related concerns in the association between continuous traumatic stress (CTS) and depression. The study participants were 499 college students who responded to an anonymous online questionnaire. Measures included the assessment of prior continuous exposure to threats of terrorism, COVID-19-related distress, perceived social support and depressive symptoms. The results demonstrated that COVID-19-related concerns mediated the relationship between continuous exposure to threats of terrorism and depression symptoms, and that perceived social support moderated the association between COVID-19-related concerns and depression. The implications of the study highlight the role of prior exposure to traumatic stress as a risk factor for depression and the role of social support as a protective factor. These results point to the need to develop accessible and non-stigmatic mental health services for populations exposed to other types of continuous traumatic stress.

## 1. Introduction

The coronavirus outbreak was recognized as a pandemic in late 2019 [1]. Morbidity and mortality rates have been increasing dramatically, with millions of deaths worldwide. In order to control the spread of the virus, authorities have been using frequent lockdowns and quarantines, which have often led to an increased sense of isolation and loneliness associated with a higher level of anxiety and depression [2,3]. The first recognized COVID-19 related stressors were fear of infection, hospitalization and death [4,5,6], intense economic concerns [7], insomnia, denial and anger [8,9], learning difficulties [10] and loneliness [11]. A review of COVID-19-related studies concluded that depression was a prominent mental effect with a prevalence of 33.7% among 44,531 subjects in 14 studies [12].

There have been various attempts to identify populations at special risk for COVID-19-related distress. Among the vulnerable populations identified were adults with co-morbid illnesses [13], minority groups [14], women [11], health care professionals [15] and university and college students [16].

Higher education students reported an increased epidemic-related anxiety, economic concerns, loneliness, loss of routine, financial concerns and limited social relationships [17,18,19]. Research based on a sample of 134,000 students from 28 countries found increased academic frustration due to changes in learning methods and the lack of clarity in teaching parameters, along with decreased motivation [20].

Although the pandemic introduced new stressors worldwide, in many cases it has added to already existing ones. Plomecka et al. [21] investigated the mental health effects of COVID-19 among 2817 participants from 12 countries and concluded that individual previous exposure to trauma was a prominent risk factor for COVID-19-related psychological symptoms, while perceived social support, optimism and daily exercise predicted fewer psychological symptoms. Seitz et al. [22] tested 85 participants in Germany and found that childhood traumatic experiences predicted an increase in PTSD symptom severity. The relationship between childhood traumatic experiences and PTSD was mediated by perceived social support but not by fear of COVID-19 [22]. However, in comparison to the vast literature on COVID-19-related stressors, less is known about the role of prior exposure to trauma in the context of COVID-19 and whether this previous and continuous exposure to stress may add to the allostatic load and accumulated distress experienced, or perhaps conversely, can function as a buffer for such distress due to habituation to stress.

### 1.1. Continuous Traumatic Stress (CTS)

The literature on CTS defines three types of exposure to trauma: a single traumatic episode (type-I), repeated similar episodes (type-II) and continuous, repeated and ongoing exposure (type-III). Eagle and Kaminer [23] argued the importance of a common yet unique type of exposure, which was continuous exposure, i.e., living everyday life under a real, present and continuous threat, such as a protracted political conflict. This last type of exposure generates CTS, in which traumatic stressors form a continuum between past and future stressors [24]. Exposure to CTS is a major risk factor for PTSD [25]. The risk can be linked to the allostatic load it can induce [26]. According to the AL model [27], perceived threats activate brain responses, leading to physiological, behavioral and psychological responses. Exposure to recurrent threats contributes to allostatic stress, inducing one of four response patterns: (1) normal/healthy, recurrent, transient response, (2) adaptation/habitation-decreased response over time, (3) prolonged response over time with no recovery, or (4) prolonged inadequate response [28]. The levels of allostatic stress depend on individual and external factors [29].

PTSD has been habitually studied as the main mental health outcome of CTS [25]. However, PTSD refers to past exposure to trauma, while CTS exposure to trauma may also be relevant to contexts in which the exposure is still ongoing and is even expected to continue in the future. CTS can be characterized by frequent changes in the severity of the stressor; it can be transient and, in most cases, it is unavoidable [25,30]. The broad phenomenon of exposure to CTS has been found to be associated with diverse emotional, cognitive and behavioral responses that are often beyond the symptomatology captured by PTSD, such as hopelessness, somatization, depression and constant preoccupation and worrying about the future [31,32,33]. Thus, Kaminer and colleagues [34] warned against extrapolation of the PTSD paradigm onto CTS and encouraged the testing of more specific distress markers such as depression, anxiety and sleep disorders [35]. Further research has suggested depression as the major stress marker [36,37,38]. Steele et al. [36] tested 972 US Navy sailors for PTSD symptoms and found that the most significant difference in PTSD screening group scores between participants below and above the PCL30 cut-off were depression scores, suggesting that depression is a major contributor to PTSD symptomatology. Logie et al. [37] studied 333 Ugandan female refugees who were exposed to life-long sexual and physical violence and found that this type of CTS predicted depression, while perceived social support predicted lower depression. Tonsing et al. [38] have linked enduring domestic violence and lack of perceived social support to depressive symptomatology among 131 Hong Kong women.

Eagle and Kaminer’s [23] conceptualization of CTS can be relevant to diverse communities including minority groups, as their group identity is linked with geographic or economic conditions. For example, migrant workers in China who encounter a multitude of practical problems have exhibited a variety of mental health problems [39]. Similarly, black people living in communities in North America who experienced life-long trauma, community violence and racism have shown increased levels of mental health disturbances [40]. Another, rather different population at risk for more severe mental health problems is combat soldiers who showed a substantial increased level of post-traumatic distress [36].

### 1.2. The Israeli Context

Israeli citizens residing within 40 km from the Gaza Strip have been exposed to continual rocket and mortar attacks for more than two decades [41], facing prolonged and ongoing life-threatening terrorism leading to CTS [42]. The consequences of living under constant life-threatening terrorism in Israel have been studied extensively [25,33,43]. Greene and colleagues [44] reviewed twenty-eight quantitative articles testing the outcomes of prolonged exposure to missile attacks among Israeli civilians. In addition to probable PTSD, they found high levels of depression, even during lower threat periods, which appeared to rise sharply during escalations in terror intensity. Others found that the prevalence of depression among CTS victims rose during threat escalation from 22.5% to 45.5% [32].

### 1.3. CTS and COVID-19

Since the onset of COVID-19 pandemic, residents of southern Israel, already living with CTS caused by exposure to terrorism, have also been suffering from additional CTS in the wake of the pandemic [45]. The question of whether the impact of COVID-19 stressors has actually augmented the stress induced by continuous exposure to political CTS is of great interest, and more specifically, whether prior exposure may serve as a risk or protective factor for pandemic-related distress. A study of the psychological consequences of the COVID-19 pandemic in Israel examined 976 Israelis, of whom 255 reported prior exposure to diverse traumatic events and terror attacks. The findings showed that previous exposure to trauma was associated with high levels of psychological distress, such as depression and anxiety [42]. Another study tested prolonged exposure to missile attacks and unemployment among 778 participants, as risk factors for emotional distress during the first COVID-19 lockdown. This research found lower resilience and higher rates of anxiety and stress among participants exposed to missile attacks in comparison to participants that had not been exposed to terror attacks [46]. Protective factors that predicted lower levels of psychological distress among participants exposed to CTS during the pandemic included living with partner(s) and being in a relationship [42], and perceived social support was associated with lower PTSD symptomatology and lower distress [47].

Social support is considered a major protective factor in the context of traumatic stress [48]. Perceived social support is defined as the extent to which people perceive that others around them are at their disposal and are attentive to their needs [49]. Perceived social support (PSS) in this study includes support from family members, friends and colleagues at college. PSS can increase people’s self-esteem, alleviate unpleasant or stressful emotions and make life comfortable and meaningful [50]. It can protect people from psychological distress after traumatic events and promote positive changes after trauma [51,52], and emotional social support can mediate the relationship between emotional intelligence and PTSD symptoms [49].

Previous study findings suggest that perceived social support could serve as a common protective factor against the psychological effects of both CTS and COVID-19 concerns, while loneliness (i.e., low perceived social support) could act as a risk factor [44,53,54].

### 1.4. The Current Study

This study focused on depression among the Israeli population that have been exposed to the CTS of terror threats for many years, and who have had to cope with COVID-19 concerns under missile and rocket attacks. Amram-Vaknin et al. [46] suggested that that CTS may be a potential risk factor for psychological difficulties during the COVID-19 pandemic. This study tests CTS as a risk factor for depression and tests perceived social support as a protective factor against depression during the COVID-19 pandemic. The paper contributes to the theoretical knowledge by suggesting and verifying a path model and by testing the empirical relationships between the factors, with theoretical and practical implications.

**Figure 1:**
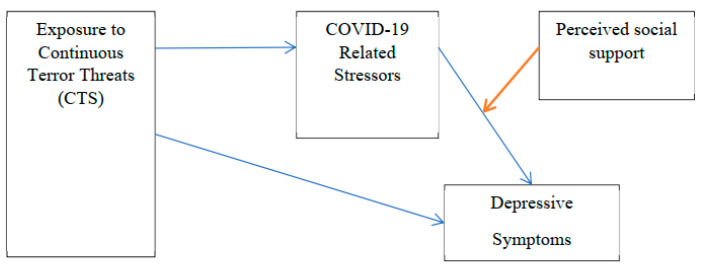
Mediation and Moderation Path Model.

Based on the allostatic load model and the centrality of social support as a potential protective factor in the context of CTS, the research aims were (a) to examine the associations between prior CTS and COVID-19-related distress with depression, (b) to test if COVID-19-related concerns mediate the relationship between prior CTS and depression and (c) to test a moderated, mediated model of the mitigating role of perceived social support (as a moderating factor) on the mediation of COVID-19-related concerns in the association between CTS and depression. Figure 1 presents the theoretical model of the moderated mediation. Based on the literature, we hypothesized a direct path between CTS and depression, with two additional paths: a mediating effect of COVID-19-related concerns on CTS-related depression and a moderating effect of perceived social support on the mediating relationship between COVID-19-related concerns and CTS-related depression.

## 2. Materials and Methods

### 2.1. Context of the Study

The study was conducted during the second wave of COVID-19 that started in early October 2020. Data collection ended after about a month. During most of that time, there were guidelines instructing citizens to “stay at home” and during a part of this period there was a total lockdown that lasted approximately 17 days. The study was conducted in a college in the south of Israel, located close to the border with Gaza and experiencing multiple occasions of terror attacks and threats.

### 2.2. Participants and Procedure

The study participants were recruited from an education academic college in southern Israel, in an area that has been exposed to continual terrorism for more than 20 years. After receiving ethics approval by the college, the Dean’s office distributed a link to an anonymous online questionnaire in Hebrew and Arabic to all students. Three reminders were sent. A total of 499 people responded. Questionnaires that were not completed in full were omitted from the sample. Therefore, only 67% of the questionnaires were included in the study. The majority of the participants (93%) reported living in southern Israel in regions that were exposed to continuous terror attacks. The sample included 90% females, explained by sampling education students and teachers, where 80% of K12 (primary to high school) teachers in Israel are women. Most of the participants were married or ran a joint household (70%) and about half of them had children (51%), with an average number of children of 2.5 (*SD* = 1.3). The ages of participants were right tail distributed (skewness = 1.03) and ranged from 20 to 73, with an average of 31.45 (*SD* = 9.49) and a median of 28. Of the total participants, 14% were studying for a diploma, 46% for a BA degree, 16% for advanced degrees and 24% were there for other studies.

### 2.3. Measures

*Exposure to prior continuous traumatic stress (CTS).* This instrument measured perceived exposure to continuous traumatic stress in the Israeli context. The participants were asked about prior exposure to four types of terrorism during the past 12 months: (1) alarms/sirens, (2) missile attacks, (3) explosions and (4) incendiary kites and balloons. Participants indicated the extent of exposure on a four-point scale of 0 = no exposure; 1 = little (a few); 2 = medium; 3 = much (a lot). We used subjective exposure rates (low/medium/high) in order to reflect participants’ subjective perceived experience. Factor analysis results indicated that the four items converged into a single index that explains 63% of the variance (eigenvalue = 2.51), and with an internal Cronbach α reliability of 0.80. The scale of exposure to terror was expressed in standard scores and calculated using a factor analysis procedure.

*COVID-19-Related Concerns.* For measuring COVID-19-related concerns, we presented nine questions to the respondents, beginning with the statement: “To what extent are you concerned about each of the following things regarding COVID…”; for example, “The situation in which anyone may infect you with the virus”. The responses were recorded on a five-point scale ranging from 1 = not at all to 5 = very much. The questionnaire included concerns about illness, infection, irresponsibility of other people, financial crises, etc. The scale was first represented and validated by Pat-Horenczyk et al. and Schiff et al. [55,56]. Factor analysis results indicated that the nine items converged into a single index that explains 59% of the variance (eigenvalue = 5.29), and with an internal Cronbach **α** reliability of 0.91. The scale of COVID-19-related concerns was expressed in standard scores and calculated using a factor analysis procedure.

*Perceived social support.* To measure social support, we used a brief form of the Perceived Social Support Questionnaire [57]. This valid and widely used scale [58] includes six items, e.g., “I receive a lot of understanding and security from others”. Responses were provided on a 5-point scale ranging from 1 “not true at all” to 5 “very true”. It should be noted that the evaluation of perceived social support was measured as a stat response, because the respondents were asked to describe their feelings these days, beginning with the statement: “These days I feel that…”. Higher scores indicate higher perceived social support. Cronbach’s α in the present study = 0.88. Similar internal reliabilities have been obtained in previous studies [58]. A composite score of perceived support was created by averaging the six items.

*Depression.* To measure depression symptoms, we used the Patient Health Questionnaire-9 (PHQ-9) [59]. The respondents were presented with nine statements, beginning with the statement: “During the last two weeks, how often have you been you been bothered by any of the following issues?”; for example, “little interest or enjoyment in doing things”. Responses for each of the questionnaire items were recorded on a 4-point scale ranging from 0 (not at all) to 3 (nearly every day), such that higher scores indicated greater depression. Inter-item reliability in present study was high (Cronbach’s α = 0.88). The ratings were summed to a total score ranging from 0 to 27. In accordance with PHQ-9 instructions, scores between 0 and 4 reflected no depression; 5 and 9, mild depression; 10 and 14, moderate depression; 15 and 19, moderate to severe depression; and 20 or higher, severe depression. A cutoff score of 10 was defined as a clinically significant level of depression, in accordance with other studies conducted during the COVID-19 pandemic [12,60]. In the present study, the median score was 6 and 30% of the respondents received a score of 10 or higher. A percentage similar to that (33.7%) has been reported by Salari et al. [12] among the general population during the COVID-19 pandemic (based on 14 studies with a sample size of 44,531 participants).

*Perceived health status.* The one-item Self-Rated Health measure was used [61], i.e., “How would you rate your health today?”, and measured with a five-point scale (1 = very good to 5 = not very good).

*Being in Quarantine.* Being in isolation due to COVID-19 was assessed by the question asking whether the participant was in quarantine and the answer options were 0 = No or 1 = Yes.

### 2.4. Analytic Plan

We first analyzed the studies variable distribution and bi-variate correlations between study variables. The hypotheses were examined using the process macro regression analysis (PROCESS Model 14) [62] to estimate the direct, mediation and moderation relationships between study variables as follows: exposure to terror served as an exogenic independent variable, COVID-19-related concerns served as the mediator and depression symptoms served as the dependent variable. Furthermore, we estimated the moderator role of perceived social support on the relationships between COVID-19 concerns and depression. Age and perceived health status were included as covariates. Process analysis allows examination of multivariate models such as mediation and moderation, using robust estimation and based on the bootstrapping technique and using 5000 bootstrap samples to assess the mediation and moderation effects [62]. Potential multicollinearity was tested and rejected, as tolerance and VIF ranged between 0.90 and 0.97 and 1.03 and 1.11, respectively. All statistical analyses were performed using the SPSS statistical package v25.0 (IBM, Armonk, NY USA) and the v3.5 process macro. Statistical threshold for significance for all measures was set at *p* < 0.05. A power analysis for detecting a medium to strong effect size (0.20) with six predictors and an α error probability of 0.01 required a sample size of 287, indicating that the current sample was sufficient for the study model.

## 3. Results

### 3.1. Correlations between Study Variables

Correlations analysis results in Table 1 revealed that depression symptoms were positively significantly associated with exposure to terror (r = 0.25, *p* < 0.001) and to COVID-19-related concerns (r = 0.49, *p* < 0.001) and negatively to perceived social support (r = −0.28, *p* < 0.001). Exposure to terror was positively significantly associated with COVID-19-related concerns (r = 0.23, *p* < 0.001) and negatively to perceived social support (r = −0.26, *p* < 0.001). Contextual status variables of age, M = 31.09 and *SD* = 9.41, and perceived health status, M = 1.54 and *SD* = 0.72, were significantly associated with the study variables; therefore, they were controlled in examining the research hypotheses.

### 3.2. Direct Effects on Depression Symptoms

We found that the total model predicting depression symptoms of students during the COVID-19 period was significant (*F*(6, 387) = 33.81, *p* < 0.001), explaining 34% of the variance in depression. Furthermore, all of the predictors had a significant direct effect on the depression symptoms. Prior exposure to terrorism, COVID-19-related concerns and perceived health status were positively associated with depression during the COVID-19 period (b = 0.58, *p* = 0.023; b = 4.67, *se* = 0.90, *t* = 5.20, *p* < 0.001, 95% CI 2.90 to 6.43; b = 1.67, *se* = 0.36, *t* = 4.64, *p* < 0.001, 95% CI 0.96 to 2.37, respectively), while, on the contrary, perceived social support had a significant negative relation with the depressive symptoms (b = −0.17, *se* = 0.05, *t* = −3.67, *p* < 0.001, 95% CI −0.26 to −0.08).

### 3.3. The Moderating Role of Perceived Social Support on the Relations between COVID-19-Related Concerns to Depression Symptoms

The effect of the interaction between perceived social support and COVID-19-related concerns on depression symptoms was significant (b = −0.11, *se* = 0.04, *t* = −2.82, *p* = 0.005, 95% CI −0.19 to −0.03). Perceived social support was found to play a role in moderating depressive symptoms associated with COVID-19-related concerns such that, when social support is perceived as low, there is a significant association between COVID-19-related concerns and depressive symptoms (b = 2.90, *se* = 0.35, *t* = 8.34, *p* < 0.001, 95% CI 2.21 to 3.58). However, when social support is perceived as high, the relationship between the variables weakens, although it remains significant (b = 1.57, *se* = 0.35, *t* = 4.46 = 3, *p* < 0.001, 95% CI 0.88 to 2.26).

### 3.4. Indirect Effects on Depression Symptoms

In addition to the direct effect of the exposure to terrorist threats on the depressive symptoms of students during the COVID-19 period, our model predicted an indirect effect of prior exposure on depression mediated by COVID-19 concerns. The analysis indicated that the indirect effect was significant (b = 0.18, *se* = 0.05, *t* = 3.76, *p* < 0.001, 95% CI 0.09 to 0.28). The partial mediating relationship between the variables was obtained at two levels (low and high) of perceived social support (b = 0.52, *se* = 0.16, 95% CI 0.21 to 0.83; b = 0.28, *se* = 0.11, 95% CI 0.09 to 0.52, respectively), although it should be noted that the mediating relationship obtained was stronger at the low level relative to the high support level. Another finding was that the covariate variables of perceived health status and age also made a significant contribution to COVID-19-related concerns, while perceived health status raised COVID-19-related concerns (b = 0.31, *se* = 0.07, *t* = 4.69, *p* < 0.001, 95% CI 0.18 to 0.45). Age was found to reduce COVID-19-related concerns (b = −0.02, *se* = 0.01, *t* = −3.48, *p* < 0.001, 95% CI −0.03 to −0.01), which later contributed, as mentioned, to an increase in depressive symptoms.

**Figure 2:**
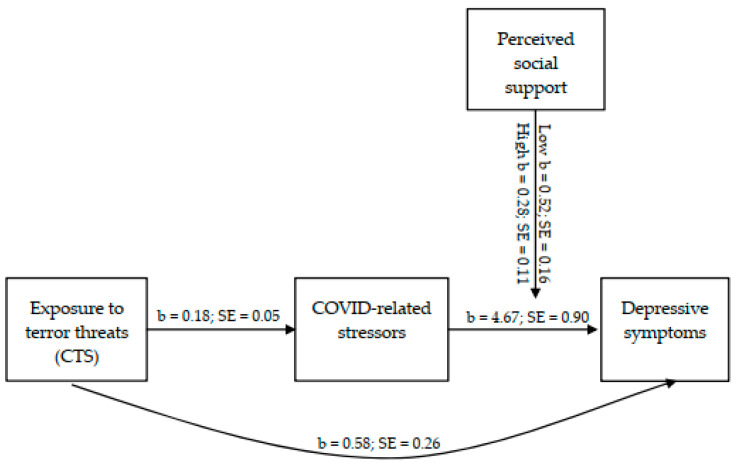
Moderating Effect of Perceived Social Support on the Relations between COVID-Related Concerns and Depression Symptoms.

**Figure 3:**
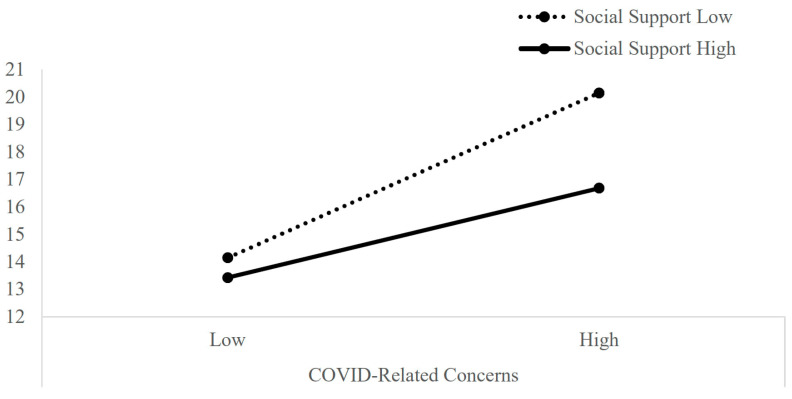
Results for the Direct, Mediation and Moderation Effects of Exposure to CST (Terror Threats), COVID-19-Related Stressors, and Perceived Social Support on Depressive Symptoms.

Full results for the direct, mediation and moderation effects of exposure to terror threats, COVID-19-related concerns, perceived health status and perceived social support on depressive symptoms can be seen in Table 2, Figure 2 and Figure 3.

## 4. Discussion

This study aimed at broadening the knowledge regarding the inter-relationships between prior exposure to continuous traumatic stress and risk and protective factors for depression symptoms in the context of the COVID-19 pandemic. We further aimed to clarify the mitigating role of perceived social support on this relationship.

It has been shown that trauma history was a risk factor for mental health problems in the context of the pandemic [42,43,63]. The study focused on the combined roles of prior continuous exposure to traumatic stress as a risk factor and perceived social support as a protective factor. Our findings support previous studies showing the association of COVID-19-related concerns with mental health problems such as depression [16], and add to the literature describing the centrality of perceived social support as a protective factor for mental distress in the face of trauma.

Our findings showed that COVID-19-related concerns mediate the effect of CTS exposure on depression. Additionally, perceived social support was also found to mitigate the relationship between COVID-19-related concerns and depression. These results support the allostatic load model that has been studied in diverse traumatic contexts [64,65,66,67]. Our findings are consistent with the hypothesis that prior and prolonged exposure to terrorism may result in increased allostatic stress and that COVID-19-related concerns might mediate this relationship, leading to higher levels of allostatic load manifesting in more severe symptoms of depression.

The “adaptive” response to allostatic load (AL) stress (pattern-1) includes repeated recovery between periods of exposure to trauma. The current study sampled the population during the second COVID-19 lockdown in Israel, but also during a relatively safe period in terms of terrorism. The participants with adaptive reactions to AL stress were assumed to be in a recovery period, thus having lower sensitivity to CTS. Furthermore, some of the participants may have developed resilience to the effects of CTS after years of exposure [46,67], displaying pattern-2 reactions. In this case, possible habitation, a phenomenon characterized by emotional adaption to prolonged threats that lowers related distress, could have occurred [68,69]. Longitudinal studies are needed in order to identify different trajectories of adaptation to CTS and additional accumulated stress.

The mediated effect of CTS was moderated by perceived social support. The ability of perceived social support to serve as a protective factor against the negative impact of stress has consistently been shown in numerous studies in the last four decades and has been supported by a large number of studies since then [70,71]. Previous studies have found that perceived social support moderates the relationships between stress and depression [45,72,73,74]. However, for the first time, to the best of our knowledge, the current study has tested the moderating role of perceived social support on the combined effect of COVID-19-related stress and the accumulation of prior and prolonged exposure to terrorism. Our findings, that perceived social support moderated the mediating effect of COVID-19-related stressors on depression among individuals highly exposed to past and continuing life-threatening terrorism, further support the centrality and essentiality of social support during mass adversity. Consistent with theory and vast empirical evidence, it was shown here again that individuals with high levels of social support from family and friends tend to be more resilient to depression when facing the accumulated stress of COVID-19 in addition to ongoing exposure to the threat of terrorism. Furthermore, perceived social support was shown to be of special importance in times of a pandemic, characterized by reduced social contacts, imposed closures and quarantines. The COVID-19 policy and regulations of maintaining social distance put many individuals at higher risk for depression and highlighted the need to find alternative ways to provide the major resource of social support and solidarity. We tested the effects of terrorism-related CTS and perceived social support on depression during COVID-19. We assumed that similar effects exist with previous exposure to other types of CTS (war, national disasters and even persistent life difficulties arising from racism, poverty, etc.) and interacting with stressors other than COVID-19. This assumption has to be tested and verified in future studies; however, the reported negative mental health outcomes of diverse CTS situations are comparable, suggesting that diagnosis and treatment might also be comparable, reflecting on professional training of therapists but also on public education programs promoting social support.

### 4.1. Clinical and Community Implications

The current study bears clinical implications for public health policy, community leaders and government agencies. Our findings highlight the role of previous exposure to traumatic stress as a risk factor for mental distress and of social support as a protective factor. This is especially important for at-risk populations who may suffer from loneliness and lack of support systems during the COVID-19 pandemic [75,76]. Both governments and NGOs are called upon to allocate financial, professional and organizational resources in order to provide infrastructure for support systems in populations at risk due to prior exposure to traumatic stress or lack of community support. Development of community services for prevention and intervention programs should gear towards both the treatment of consequences of exposure to CTS and strengthening support on all levels (personal, familial community and cultural).

Workplaces, academic institutions and other community organizations should acknowledge the importance of social resources as a means to improve the mental wellbeing of workers and students, and to facilitate new venues for such support in the occurrence of a pandemic. Providing, enabling and financing such services could also increase the trust that individuals and groups have in their governments, municipalities, employers and organizations—a trust that is most needed in times of turmoil such as those that the COVID-19 pandemic has brought to the world. Obviously, there is a need to develop accessible and non-stigmatic mental health services with a special emphasis on enhancing social support for populations exposed to prior continuous traumatic stress. Finally, our findings stress the need to integrate mental health specialists into COVID-19 services.

### 4.2. Limitations and Future Research

Our study has several limitations. First, this study was based on an online survey of self-reporting questionnaires. Second, the population was tested during a second COVID-19 closure, in a period of decreased terror attack activity. Third, the sampled population included only students, and most of them (90%) were women due to sampling in an education college, effectively sampling a specific population with possible specific characteristics that might have skewed the results. The sampling bias clearly limits generalization of the results, and further studies are required in order to test the generalizability of the moderated, mediation model, using non-biased sampling in diverse populations and environments, including exposure to other types of CTS. Further research should test the combined effects of CTS with other acute stressors as well as the combined effects of multiple CTS sources. To extend our understanding, future research should also include cross-sectional studies that compare different social/ethnic groups and cultures and diverse CTS types using the same measuring tools. Finally, longitudinal research is required to test causality of the effects and relationships between research variables.

## 5. Conclusions

Exposure to continuous traumatic stress can lead to psychologic difficulties, with depression as one of the major negative outcomes. Our findings demonstrate that previous exposure to terror-related CTS can increase COVID-19-related stress and increase COVID-19-related depression. Perceived social support could decrease COVID-19-related depression and minimize the negative effect of CTS during COVID-19. It is safe to assume that other types of CTS have similar effects and are similarly affected by social support, yet further studies are required to support this assumption. However, the findings are important to governments, institutes, NGOs, community aid agents, therapists, caregivers, educators and individuals. Simply said, organizations, groups, communities, families and individuals should recognize the positive effects of social support and act upon this knowledge to support others against the negative effects of CTS, COVID-19 and possibly other stressors. We also conclude that additional research is required to test and compare the practical implications of exposure to diverse sources of CTS and stressors in different demographical, political, cultural and socio-economic environments.

## Figures and Tables

**Table 1:** Descriptive Statistics and Correlations for Study Variables (N = 393).

		M%	*SD*	1	2	3	4
Study variables	1. CTS *^a^	0.03	1.03	-	0.23 ***	−0.26 ***	0.25 ***
	2. COVID-19-related concerns ^b^	0.01	1.01		-	−0.15	0.49 ***
	3. Perceived social support	22.13	5.62			-	−0.28 ***
	4. Depression symptoms	16.13	5.96				-
Contextual status	5. Age	31.09	9.41	−0.24 ***	−0.21 ***	0.21 ***	−0.17
	6. Gender ^c^	90%	-	0.08	0.11	0.07	0.18
	7. Quarantine (yes)	46%		−0.07	−0.13	0.07	0.01
	8. Perceived health status	1.54	0.72	0.04	0.22 ***	−0.17	0.33 ***

*Note:* * CTS = (continuous traumatic stress—exposure to continual terrorism). ^a,b^ Z scores. ^c^ 1 = female and 0 = male. *** = *p* < 0.001.

**Table 2:** Results for the Direct, Mediation and Moderation Effects of Exposure to Terrorism, COVID-19-Related Concerns, Perceived Health Status, Age and Perceived Social Support on Depressive Symptoms.

	Estimate	*SE*	*t*	*p*	95% CI	
					*LL*	*UL*
Model 1: Depressive symptoms						
Intercept	18.27	1.41	12.91	0.000	15.49	21.05
Exposure to terrorism	0.58	0.26	2.29	0.023	0.08	1.09
COVID-19-related concerns	4.67	0.90	5.20	0.000	2.90	6.43
Perceived social support	−0.17	0.05	−3.67	0.000	−0.26	−0.08
INT*	−0.11	0.04	−2.82	0.005	−0.19	−0.03
Perceived health status	1.67	0.36	4.64	0.000	0.96	2.37
Age	−0.03	0.03	−1.26	0.210	−0.09	0.02
*F*(6, 387) = 33.81, *p* < 0.001; *R*^2^ = 0.34						
Model 2: COVID-19-related concerns						
Constant	0.10	0.20	0.50	0.618	−0.29	0.48
Exposure to terror threats	0.18	0.05	3.76	0.000	0.09	0.28
Perceived health status	0.31	0.07	4.69	0.000	0.18	0.45
Age	−0.02	0.01	−3.48	0.001	−0.03	−0.01
*F*(3, 390) = 18.97, *p* < 0.001; *R*^2^ = 0.13						
*Indirect Effects thru perceived COVID-19-related concerns moderated by social support levels*						
Low social support	0.52	0.16	-	-	0.21	0.83
High social support	0.28	0.11	-	-	0.09	0.52

*Note: N* = 396. Bootstraps Sample Size = 5000. CI = confidence interval; *LL* = lower limit; *UL* = upper limit. For examining the interaction, a transformation was made to the quantitative variable, perceived family support, to qualitative, when the cut points were above and below 16th and 84th percentiles. INT* = interaction between COVID-19-related concerns × perceived social support.

## Data Availability

Data are available upon request from the first author, Beck Leshem, at beckyleshem@gmail.com.
